# Nurses’ perspectives on supporting children during needle-related medical procedures

**DOI:** 10.3402/qhw.v9.23063

**Published:** 2014-03-12

**Authors:** Katarina Karlsson, Ingela Rydström, Karin Enskär, Ann-Charlotte Dalheim Englund

**Affiliations:** 1 School of Health Sciences, University of Borås, Borås, Sweden; 2 Department of Nursing Sciences, CHILD Research Group, School of Health Sciences, Jönköping University, Jönköping, Sweden

**Keywords:** Lived experience, caring science, younger children, phenomenology, reflective lifeworld research

## Abstract

Children state that among their worst fears during hospitalization are those related to various nursing procedures and to injections and needles. Nurses thus have a responsibility to help children cope with needle-related medical procedures (NRMP) and the potentially negative effects of these. The aim of the study is to describe the lived experience of supporting children during NRMP, from the perspective of nurses. Fourteen nurses took part in the study, six of whom participated on two occasions thus resulting in 20 interviews. A reflective lifeworld research approach was used, and phenomenological analysis was applied. The result shows that supporting children during NRMP is characterized by a desire to meet the child in his/her own world and by an effort to reach the child's horizon of understanding regarding these actions, based on the given conditions. The essential meaning of the phenomenon is founded on the following constituents: developing relationships through conversation, being sensitive to embodied responses, balancing between tact and use of restraint, being the child's advocate, adjusting time, and maintaining belief. The discussion focuses on how nurses can support children through various types of conversation and by receiving help from the parents’ ability to be supportive, and on whether restraint can be supportive or not for children during NRMP. Our conclusion is that nurses have to see each individual child, meet him/her in their own world, and decide on supportive actions while at the same time balancing their responsibility for the completion of the NRMP. This work can be described as “balancing on a tightrope” in an unpredictable situation.

All children have to endure a number of medical procedures through childhood and adolescence (Blount, Piira, Cohen, & Cheng, 2006; Power, Liossi, & Franck, 2007) such as injections and needles. It is common that children experience fear and pain (Gaskell, Binns, Heyhoe, & Jackson, 2005; Melhuish & Payne, 2006; Meltzer et al., 2008), and if the children have a long-term disease, the risk is that they will be exposed to recurrent needle-related medical procedures (NRMP) (Blount et al., 2006; Power et al., 2007).

This is the first study of four in a larger project aimed at generating knowledge about the various aspects of how children experience and cope with NRMP and the support provided for them. The larger project includes the perspective of the child, the parents, and the nurses. This article focuses on the lived experience of supporting children during NRMP, from the perspective of nurses.

## Background

It is essential for nurses to have knowledge about children's experiences of hospital-related fears (Salmela, Salanterä, & Aronen, 2009). In order to be able to support children, it is important that nurses have an understanding of what fear stands for, and how children communicate their fears (McGrath & Huff, 2001; Rennick, Johnston, Dougherty, Platt, & Ritchie, 2002). Previous studies with younger children are mainly based on adults’ perceptions of what children consider to be painful and frightening (Kortesluoma & Nikkonen, 2006), but studies on how young children themselves describe their fears have now also been performed (Salmela, Aronen, & Salanterä, 2010; Salmela, Salanterä, Ruotsalainen, & Aronen, 2010). Fear experienced by children could, for example, be augmented as a result of being in an unrecognizable environment, in terms of equipment and unfamiliar people (Lindeke, Nakai, & Johnson, 2006; Salmela, Aronen, et al., 2010; Salmela et al., 2009) and also separation from their parents (Salmela, Aronen, et al., 2010; Salmela et al., 2009; Snyder, 2004). Children can express their fear in different ways, such as not talking much, or withdrawing, but also describing their fears in some detail (Anderzén Carlsson, Sørlie, Gustafsson, Olsson, & Kihlgren, 2008). Previous studies show that children in all age groups are worried prior to a medical procedure, but that younger children experience a more diffuse fear and feeling of uneasiness (Hedström, Haglund, Skolin, & Von Essen, 2003). The staff helps children in different ways during medical procedures. This support can include making the unknown known, being sensitive, and by distracting the children (Anderzén-Carlsson, Kihlgren, Skeppner, & Sørlie, 2007).

Children state that among their worst fears during hospitalization are those related to various nursing interventions (Lindeke et al., 2006; Salmela, Aronen, et al., 2010; Salmela et al., 2009), such as being exposed to injections and needles (Kettwich et al., 2007; Salmela et al., 2009), and the needle thus symbolizes a strong negative feeling. Uman, Chambers, McGrath, and Kisely (2006) explain NRMP as an investigation or action that children have to endure in order to prevent illness, to enable diagnosis, and to give treatment and that involves the use of needles. Furthermore, Noel, McMurtry, Chambers, and McGrath (2009) state that if children are subjected to high levels of pain intensity instantaneously after the procedure, they often experience excessive feelings of anxiety and pain later on in life and can also develop pessimistically exaggerated memories. Children, who have once developed exaggerated memories, can develop problems in the future when exposed to NRMP. Children's negative memories of pain and anxiety early in life can lead to needle phobia and also desires to avoid medical care later on in life. This is also discussed by Von Baeyer, Marche, Rocha, and Salmon (2004) and by Walco (2008) who write that children's suffering related to procedural pain may result in changes in their pain systems that also can lead to problems in the future. Taddio et al. (2009) discuss that untreated pain can lead to fear of needles.

One of the duties of a nurse, as part of the ethical conduct, is to function as the patient's advocate (Balwin, 2003; MacDonald, 2007). Nurses also have four essential responsibilities: promoting health, preventing illness, restoring health, and alleviating suffering, which are quality requirements for Swedish registered nurses (Swedish National Board of Health and Welfare, 2005, p. 17). Furthermore, a part of a nurse's work is to ensure that the patient's pain is minimized (McCabe, 1997), and in the case of a child, one additional important aspect is to relieve the child's anxiety and worry because these experiences can intensify their feelings of pain (Wood, 2002).

Consequently, nurses have a responsibility for supporting children to cope with NRMP and any potential negative effects (Melhuish & Payne, 2006; Wood, 2002). There are a number of different strategies for helping children during NRMP and Blount et al. (2006) divide actions into the use of medical, psychological, and merged activities, whereas the review by Uman et al. (2006) shows that nurses can support children by using hypnosis, distraction, and cognitive behavioral therapy.

Although research has been carried out on nurses’ supportive function when children undergo medical procedures, there seems to be a gap in literature related to the lived experience of supporting children at NRMP, from the perspective of the nurses. This study can contribute to the comprehension of nurses’ experience of supporting children and also to care development for those children who have to undergo these procedures. The aim of this study is to describe the lived experience of supporting children during NRMP, from the perspective of nurses.

## Method

### Design

In order to describe the phenomenon, supporting children during NRMP, reflective lifeworld research (RLR) was used and a phenomenological analysis was applied, as described by Dahlberg, Dahlberg, and Nyström (2008). RLR is grounded in lifeworld phenomenology and caring science and is based on the philosophical ideas of Husserl (1950)/1977), Heidegger (1962)/2008), and Merleau-Ponty (1945)/2002). RLR contains three central concepts: openness, sensitivity, and bridling (Dahlberg et al., 2008), which were used throughout the study. This generally means an ability to listen as a researcher with an awareness of what nurses’ experience. Openness refers to a genuine desire to see, hear, and understand that in this case relates to nurses’ experiences of NRMP. Being open thus entails being responsive towards a phenomenon and at the same time being sensitive. To bridle can be understood as an effort to hold back pre-conceptions, which in this study is our pre-understanding about supporting children during NRMP. The study was conducted by using video-recorded observations from NRMP with subsequent interviews.

### Settings

The study took place at four different units: child health care services, pediatric primary care services, pediatric inpatient care, and pediatric outpatient care, in a county in the southwest of Sweden.

### Participants

Fourteen nurses gave informed consent and took part in the study. The nurses were recruited during a workplace meeting or were asked to participate on the same day of the data collection, on both occasions by the first author. Inclusion criteria were that the nurses during the study period had participated in one or two NRMP with children aged 3–7 years with non-acute or life-threatening illness, that they were willing to participate, and that the child and parents had given informed consent. Eleven were pediatric nurses, two were general nurses, and one had another specialist education. The nurses had worked in healthcare for a mean of 25 years (range 9 months–42 years) and as nurses, 18 years (range 9 months–31 years).

### Data collection

The data collection was conducted from the spring of 2011 to the summer of 2012 and included 20 meaning-oriented interviews (Dahlberg et al., 2008), accompanying 20 video-recordings of the NRMP. Six of the nurses participated twice, but in order to facilitate a variation of the phenomenon, supporting children during NRMP, it was considered best to restrict each nurse's participation to just two occasions.

The NRMP included in the study were skin tests for allergy, blood sampling (venous or capillary), intravenous cannula insertion (IV), needle insertion in a central vein port, and injections into the joint. All children were given standard therapy for NRMP which includes some form of topical anesthesia, apart from capillary blood sampling and skin tests for allergy. The topical anesthesia was applied at least 1 h prior to the NRMP. Standard therapy was also used with inhalation/sedation, N_2_/0_2_, for children who underwent injections into the joint and for those who had a needle phobia.

All NRMP were video-recorded by the first author, to be used as stimulated recall during the interviews. After the NRMP were completed, the time and location for the interviews was chosen by the nurses and took place individually at the nurses’ workplace, directly connected to NRMP or in the days after. The interviews began with an opening question “Would you like to tell me about your experience supporting children during NRMP?” Follow-up questions such as “Can you tell me more?” and “How do you mean?” were then asked. The aim of this type of questions was to encourage in-depth reflection and to gain descriptions of the variations in the experiences of supporting children during NRMP. This means that in order to gain a deeper understanding of the phenomenon, the nurses began to describe the specific procedure and then talked more generally about their experiences of supporting children during NRMP. The video-recording from the NRMP was shown in order to further increase the opportunity for reflection. All the interviews were recorded on a MP3 player and were transcribed verbatim by the first author. The mean time for the interviews was 29 min (range 9–55 min) and for the video-recordings from the NRMP 11 min (range 4–30 min).

### Data analysis

Data from the meaning-oriented interviews were analysed using RLR and a phenomenological approach according to Dahlberg el al. (2008) which aims to find descriptions based on the phenomenon: supporting children during NRMP. The characteristic for the analysis is a constant movement between the whole and the parts. The data analysis began by reading the interviews in their entirety to become familiar with the text, and at the same time trying to have an open and reflective approach in order to get close to the text, without starting the analysis process. When the text appeared familiar as a whole, the next phase, where the text was divided into parts, termed meaning units, began. These were marked in the text and described with a few words. Those markings formed different related clusters, each one consisting of meaning units that linked to the others. A new whole revealed from different clusters when all the meanings had been identified, forming a description of the essence of the phenomenon, i.e., of supporting children during NRMP. The essential meaning was formulated and further described by its constituents, which are nuances of the essence. During the entire analysis process, there was a constant movement back and forth in the text, in order to ensure the validity of the findings.

### Ethical considerations

Ethical approval was obtained from the Regional Ethical Review Board of Gothenburg (Dnr 724-10). The Helsinki Declaration (2008) was followed. The permission to conduct research was also given by the director and the head nurse from each department. The nurses were recruited in two different ways: by receiving prior information during a workplace meeting or by being asked to participate the same day of the data collection, on both occasions by the first author. The nurses were informed that their participation was voluntary and confidential. All participants were informed that the NRMP would be video-recorded. Additional information was provided orally and in writing. The first author was waiting for participants on the unit where the nurses worked. This could entail the nurses feeling pressure to participate, when seeing the first author on a few occasions. The potentially negative effect of this was reduced by the first author providing information and requesting their participation on only one occasion, during the same working shift.

## Findings

The essential meaning of supporting children during NRMP is characterized by a desire to meet the child in his/her own world, and an effort to reach the child's horizon of understanding regarding these actions, based on given conditions. This requires sensitivity towards the child's health status and towards the current living situation, including existential issues. Meeting the child's needs here has the character of “balancing on a tightrope” through an unpredictable situation. Part of the unpredictability is related to the time aspect, which can either assist or obstruct the implementation of the NRMP. The meeting should also incorporate an openness to the child perspective. Most importantly, it will require a repertoire of supportive activities in which parental presence and conversation are the cornerstone. The essential meaning shows that parental presence plays a central role in the child's feeling of security. However, parental participation must be guided by an awareness of the child's needs and of the parent's ability to provide security. Parents can be seen as an extension of the child in order to facilitate access to the world. Through conversation, which must be conducted with tact, the child will be involved in what is going to happen. This will not only provide security, but also generate interest. Specifically, it is about “interpreting what the child expresses” and using one's instincts to choose the right words in the dialogue with the child. The conversation is thus adapted to the child's state of mind, experience, and age. The conversation is combined with play, which can be of a wide variety of different types and can fulfill different functions. This play is most importantly a tool for approaching the child's understanding and world of experience. Through play the child is given an active role and control of the situation, which distracts and de-dramatizes the current NRMP.

As the context and atmosphere of healthcare situations are unfamiliar, it can intimidate the child. The child's previous experiences of healthcare are crucial here as it relates to their experience of the situation, and subsequently how dangerous it may feel to them. The situation disrupts the child's world and provides lasting memories, which should be actively influenced in order for them to become positive.

The essential meaning thus shows that the interpersonal meeting requires a tactful interaction between all people involved. In this meeting, quick and spontaneous decisions are made that allow the balancing act to be maintained. The basis for this meeting is knowledge and an ability to understand the world in the way each individual and unique child experiences it. This is balanced with the given conditions that are necessary for the implementation of the NRMP.

The meaning of the phenomenon of supporting children during NRMP consists of the following six constituents that describe the variations and nuances of the phenomenon: developing relationships through conversation, being sensitive to embodied responses, balancing between tact and use of restraint, being the child's advocate, adjusting time, and maintaining belief.

### Developing relationships through conversation

One way of supporting children during NRMP is through conversation. Conversation concerns the provision of information, making “small talk,” conversing through others and conversing using simple words and metaphors. Nurses maintain that the information provided for the child needs to be balanced so that it does not inhibit action.

Supporting children requires an adjustment to the amount of information the children should receive, based on their age, illness, degree of participation, experience, fear, and ability to focus. Younger children at the age of 3 to 4 years, with no prior experience of NRMP, will thus receive limited information; similarly children with more experience will be getting more detailed information. Nurses also explain that children who are anxious receive less information while open and curious children can be given more explanation.

Making “small talk” is one way of calming the child while talking about something else other than NRMP. Nurses suggest that the person who makes the “small talk” is someone who the children decide on although most nurses want to do it themselves: *Often when we get started with the procedure parents become tongue-tied. Then I have to go in and talk anyway*. Additionally the nurses state that for very sick children “small talk” seldom provides a means for distraction, although information remains equally important.

Using one to reach the other occurs when nurses talk with the children to reach the parents and sometimes vice versa:

I speak as much with the parents actually, though I always talk to the child … But I always show the plastic tube first and I show it to the parents … We have such short meetings so we need to create trust in each other first. Parents feel it [plastic tube] and the parents give it to the child.

In order to be able to support children through conversation it is important to choose the right expression so that children understand what is going on and not experience fear; otherwise the NRMP becomes more difficult to perform. The nurses’ level of experiences, as well as the age and experience of the children are helpful when choosing words and phrases that fit the moment. One way to conceptualize this is to make use of helpful metaphors, as parents and even children do, and are described by an nurse like this: *A child says while the patch is removed ‘it's as slow as a snail when it goes’*. Another example of how nurses use metaphors: *I think it's like a small aircraft … You can refuel … You can say ‘the airplane has crashed’ [if the intravenous cannula insertion fails] … It has worked well for what I call ‘a pvk’ [cannula] which doesn't mean anything to them*.

### Being sensitive to embodied responses

It is important for supporting children during NRMP that the nurses are sensitive to the body language expressed by children and parents as well as its meaning.

Nurses can get a sense of whether the children and their parents feel secure or not, prior to the NRMP, by reading their signs. The time is shared based on the greatest immediate need of support, the child or the parents, and on the nature of the support that is needed. This requires quick thinking and sensitivity to what children say and express with their face and body. A child's bodily expression can be a useful tool for understanding the meaning, rather than solely using the spoken word: *I think it's horrible with kids when you notice that they are really, really scared, but they don't dare to say it*. Nurses also read the parents’ expression for determining whether they are safe enough to support their children during NRMP.

Support by being responsive entails the experiences of both the children and the parents being seen and alleviated. The nurses interpret the body language of the children prior to the procedure, and if the expression of the children indicates that they feel secure, then the nurses ask them if they want to watch what is going to happen. On the other hand when children display insecurity then such a question will not be asked, and instead nurses say *this is how we usually do it* and screens it off with a book. Children may react with fear at seeing, for example, the needle coming towards them: *If they see the needle and it arouses the Amygdala [center in the brain] and they naturally withdraw themselves from any danger. These are things that come natural to us*. If children see the needle and react with fear the nurses must postpone the NRMP.

In order to support the children, it is important to be able to distinguish between fear of NRMP and resistance to it being done. The children who are scared show no sign of curiosity and it makes it difficult for the nurses to proceed. However, if the children oppose the action, the nurses can work with them to convince them. When the children are younger it is often difficult to determine their ability to watch what is done despite their curiosity: *Some children can be very curious and then when they see the needle and it's time for the blood test they freak out*. Nevertheless, it is difficult to determine the difference between fear of NRMP and resistance to it being done, and it requires sensitivity on behalf of the nurses to make the right judgment.

Children, who come for repeated NRMP, develop their own ways of dealing with the action. The way the children express themselves thus helps the nurses to differ between perceived pain and fear. Pain manifests itself in the way children jerk and scream at the moment of the needle prick. Fear is similarly often manifested through screaming: *She screamed more before the stick*. Nurses state that if they can distinguish between children's pain and fear, it will help them to choose the appropriate action to support the children during the NRMP.

In order to support children, nurses try to interpret and to understand which environment is best for the children when the NRMP is to be performed. If the children are hospitalized, the procedure can be carried out in their room. There are both advantages and disadvantages for this setting, as the children have their own things there and a nice, soft bed, but on the other hand nothing scary should be performed there. There is equipment in the treatment room that children can perceive as frightening, according to nurses, although everything is available there.

### Balancing between tact and use of restraint

Approaching children tactfully at the time of the NRMP entails adapting to each individual meeting so that the child's health and the perception of the situation are taken into account. Nurses use play in order to support children in a tactful manner. In certain situations, tactfulness is tested, for example, when restraint becomes necessary.

In order to be able to support children the nurses must become acquainted with them, and this should be performed in a considerate way. When the nurses acquaint themselves with the children, the former assess how children feel and how they experience the situation and this is relevant for how smoothly the NRMP goes: *Very sick actually, and very sad, and his general state of health was not good at all … so he prolongs the whole process*. Children who need to experience repeated NRMPs often become accustomed to them. However, if this is not the case, the procedure becomes even more difficult over time:

He does not want to have it, but he's got to … It is not easy for a small boy; he's had to put up with a needle stick once a week and it hurts … I think it's become worse over the years.

Play is of great importance when it comes to meeting children, in order to be able to tactfully engage the children and give them support during the procedure. Play is incorporated into the procedure in order to make the preparations understandable for the child, which thus increases the chance of a successful outcome. For example, the children can look and feel the equipment. This involves trying it on a doll, teddy bear, parents, or staff. To support the children during the action and enable better processing afterwards, children can bring materials home to play with. Similarly such material can be helpful in preparing the children. Nurses state that distraction in terms of play is also a focal point when supporting children. This often helps to make the situation less threatening and helps the children to think of other things during the procedure: *You want to play it down, so you're a bit of both, a clown and a nurse*. Children who are worried and frightened need to be distracted when the procedure is performed. At such times it is important when there are two members of staff where one distracts and the other performs the NRMP. For the nurse who performs the action, it is important to be neutral: *Don't go on and smile and think what fun this is. For the child, this isn't a funny situation. But then I don't need to look as though I'm strict or angry*. The nurses believe that it is best for the children when distraction is not needed. If the NRMP is performed without distraction, the children gain more knowledge about the action until the next time.

Children do not always willingly participate in the NRMP despite preparation and distraction. This thus puts tactfulness to the test and restraint may become necessary. In these situations the nurses also need to find a way to help the children, which can include preparing them with sedative drugs, anesthetic patches, or nitrous oxide. Nurses say that it is important that parents give their consent and that the children, as far as it is possible, are involved in how the procedure is to be performed: *You need to have parents with you, because you can't oppose both the children and parents*. Nurses describe that the loss of control that restraining can result in is even worse than the syringe: *They've just been held down and it becomes an injustice, that's what it's like*. In spite of this, restraining a child is sometimes necessary, and is thus at the same time supportive, because scared children can build up an internal stress: *Allowing a child to decide for him/herself isn't good because then we would have been sitting there discussing maybe for half an hour, 40 minutes*. While one nurse says that: *For my own sake, it might be easier and just go in and do it quickly. But it isn't good in the long run*. The children's previous experience of care is relevant for how well the procedure goes. According to one nurse, a mother spoke of them having experienced a difficult situation at the child health care services the previous week, when four people had to restrain the child while taking a blood sample, and the nurse says: *If there's been a situation like this, then you must handle it a little differently*. An interruption can, in certain circumstances, be supportive in that the child can go home and prepare for another occasion. If the children have no previous experience of NRMP, they can feel fear because the procedure is not familiar and they thus can be helped by being held: *Then they are afraid because they don't know what will happen. And they are afraid of losing control*.

### Being the child's advocate

Parents are first and foremost the children's representatives, but if the parents are unable to do this, the nurses will take the responsibility for supporting children during NRMP.

It requires at least one parent for the procedure to be carried out. The nurses help and guide the parents if the latter for some reason are unable to support the children. If the children are calm and if they feel safe the supportive action is less important, the role of the parents thus automatically becomes less prominent. On the other hand, however, if children are scared and worried, it requires greater involvement from the parents. When parents act the nurses are in the background:

I think that it can be an advantage when a parent goes in and I back away, a little … If you have one who is not really able to hold or just lets go. There'll be no support for the child either and you just pass on more concern to the children. And then the procedure just gets more complicated.

Providing support for the children during procedures can also mean that the nurses have to represent the child: *Sometimes you have to take the* *child's* *part* *and strengthen them* … *‘It doesn't hurt’ as some parents say. I usually say ‘it does’ … Never try to fool the child and say that they won't be feeling anything*. The nurses are also the children's representatives when contact with the physicians is concerned. The nurses thus ensure, among other things, that all ordinations have been prescribed so the children will not be exposed to a needle prick twice that day, or as described by a nurse like this: *Then you have to be the voice of the child’ quite simply. There are doctors who prescribe lots of tests. What in the world, we can't just take that amount of blood*. Nurses specify that physicians are rarely present at NRMP and therefore do not see if the children have a difficult time: *We* *often* *tell* *doctors* *that it is hard* *with* needle *sticks* …. *That you* *can't*, *wait* *several months and* *the child will* *suffer* … Be*cause we*, *who are nurses*, *most often see* *immediately* *the need for* *a port* *á* *cart*.

### Adjusting time

Time is of great importance when it comes to supporting children during NRMP. More specifically it is about being able to get the right amount of time for each individual child.

NRMP with children takes time, but for some children additional time might impair the implementation and thus inhibit support: *She cannot* have *that extra* *time that* *many children* *want and it* *almost* *becomes bothersome*. *It has to be done anyway*, *they* *just* *get worked* *up*. Procedures that are performed acutely appear to increase the fears of the children while the planned NRMP reduce it as the children have time to prepare.

Timing proves essential when supporting the child through reducing fear and increasing control. While time can be saved by allowing parents to put on a topical anesthetic patch at home, it can also be perceived as a disadvantage, because children often need time to familiarize themselves with the new environment: *I often think that they are helped as soon as they are given local anesthetic and may be in the playroom [at the hospital] and during the time for the local anesthetic to work they play and feel more at home*.

Nurses have to support the children in cases were parental behavior is influenced by a lack of time. For example, when the parents have little time for the topical anesthetic to be effective, the nurses takes over as much as possible: *If the parents say, ‘we don't need the local anesthetic, our parking time is going out’. Then they'll have to come another day*. For the benefit of the child nurses occasionally blame their lack of time to delay the procedure by the physicians. The nurses maintain that a lack of time should not negatively affect the children or the parents during the procedure.

### Maintaining belief

Giving hope and courage is one way of supporting children. It also entails praising the children after the NRMP so that they have positive experiences to take with them for future visits.

When being supportive the nurses use words such as “brave” and “good” when talking to the children. When using “brave,” the nurses find that the children want to participate and have an understanding that the NRMP must be carried out, even if they are unable to participate during the whole procedure. The nurses say that the word “good” is incorporated in difficult procedures where the child is sad.

While the nurses prefer using the word “brave” the two concepts appear to be used randomly, without distinguishing their specific meanings. To be brave implies that the children have coped with something unpleasant: *So, you were so brave, you still did it despite being sad and not wanting to do it before, and you did it anyway. I think it's important to communicate this so the principle of being good does not prevail* [principles described by nurses].

Another way of being supportive is to allow the children, prior to the procedure, to look in the gift box. While gifts are primarily given to the children who undergo NRMP, siblings occasionally also receive a gift: *So, if the patient, who I took a sample from really wanted a sibling, who has been involved and supportive, wants to share something, then I feel, that's OK*. It is important that all staff think in the same way about who should get the gifts: *It should be the same for everyone* *so* *that it is not ‘when you come to this nurse it will be like this and when you come to another nurse’, then it will be like that*. *G*ifts have a dual function, in relation to NRMP: *I also believe they are important to me as a caregiver who does this with the child. I mean, after all, however hard I try it will be experienced on many occasions as an injustice*.

Furthermore the nurses notice that if the NRMP has been difficult for the children, they seldom accept gifts. Occasionally the gifts are also seen as bribes by the children. In situations like these, nurses provide the parent with gifts so that the children can receive them when they are at home and are feeling more secure. The act of bribing is, according to the nurses, done by nurses and parents, a procedure that will enable them to reach the children and carry out the NRMP more smoothly: *And so we're bribing them quite a lot with ice cream and sweets and little things like that*.

Most importantly, nurses must sometimes support the parents in order to help them support their children. The nurses state that the parents might react negatively if the children are yelling and refusing to be still during the NRMP and the nurses must then support the parents.

This is done by the nurses talking to the children so that the parents can hear them: *They* *may* *feel* *that the child* is *screaming* *unnecessarily*. *But* you must *always* encourage, so *it's* *okay to* *be upset*, it's okay *that it hurts*.

If the NRMP fails, the nurses must be supportive and provide hope and courage for the next time. In this context, a failure might include the child crying and being unable to be still, which can result in them not being satisfied with themselves. Yet, how the nurses inspire hope and courage can be difficult to put into words: *It's really hard to explain. So, there's this diffuse feeling that you have in your fingertips that you can't really explain*. Nurses mean, however, that this kind of embodied knowledge is something that can be gained through years of experience.

## Discussion

### Reflection on the findings

This study was undertaken to describe the lived experience of supporting children during NRMP, from the perspective of nurses. The analysis resulted in the following constituents: developing relationships through conversation, being sensitive to embodied responses, balancing between tact and use of restraint, being the child's advocate, adjusting time, and maintaining belief; and the discussion will focus on some of these findings.

As Mohr (2010) claims, the decisions that nurses make in their work must rest on four ethical principles: autonomy, beneficence, justice, and non-maleficence. Mohr implies that their work can be seen as creating therapeutic relationships between all those involved in the caring process. This is highlighted in our study and the therapeutic relationship is thus found between the children, the parents, and the nurses.

The findings indicate that nurses use different types of conversation in their attempt to be supportive when talking to children and their parents. Metaphors can be used to facilitate an understanding between the child and the nurses, helping the child to become involved in the procedures. Most importantly, the nurses are able to talk in a language that the child understands. This finding is consistent with previous research from Kortesluoma and Nikkonen (2006) who maintain that children from the age of five are able to construct metaphorical expressions. Fleitas (2003) also discusses the benefits of using metaphors when talking with children in pediatric settings. We believe that nurses can be supportive by using metaphors although nurses have to be vigilant as there is a risk that children do not always understand, especially the younger children.

The nurses in our study also speak of the difference in content between providing information and making “small talk,” where the latter is not focusing on the actual procedure. Plumridge, Goodyear-Smith, and Ross (2009) differ slightly in that “small talk” is based on professional skills and can, for example, mean that nurses are talking to children about the procedure and provide clues with information meant for the parents. Similarly, a study presented by Mahoney, Ayers, and Seddon (2010) refers to non-procedural talk (which can be assumed to correspond to “small talk”) being the most frequent coping behavior in school-aged children, parents, and healthcare professionals. This is consistent with our study in that talking and giving information to someone other than the referred person is something that nurses do in different contexts. We also believe that it is important to distinguish “small talk” from information in that the former should include everything concerning other matters than the actual procedure, in order to help the children to think about something other than the NRMP. We thus believe that “small talk” is just as important as basic information.

Findings from the present study also show that being the child's advocate entails guiding the parents in being the child's representative as well as protecting and representing the child when the parents’ support is not sufficient, for example, when the parents may fear that the NRMP will hurt their child. Schechter et al. (2007) and Cohen, Manimala, and Blount (2000) maintain that non-helpful responses from parents towards the child can increase the stress in children during procedures. Examples of non-helpful responses include being extremely reassuring, criticizing, using excuses, and being empathetic. Schechter et al. (2007) also state that children's distress increases in relation to the degree of involvement from parents. It is interesting to note that, according to the review by Boudreaux, Francis, and Loyacano (2002) and the study by Waseem and Ryan (2003), health professionals’ perception of parents’ presence at different medical procedures varies greatly. This does not concur with the results from our study where nurses felt that parents should be present but that their “duty” could be different depending on their abilities. We thus state that a crucial task in the support to the children is the provision of the necessary information to the parents in order to enhance their involvement. This will facilitate a change of focus from protecting the child from the nurses to supporting care. Sometimes the nurses will take over and act as the child's advocate by supporting and representing the child. This must be done in a way that is not perceived shamefully for any of the involved and requires a sensitivity from the nurses. Once again it is the nurses’ ability to balance the diverse needs that is the key to the quality of care in order for the dignity for all involved to be maintained. MacDonald (2007) concludes that nurses have both an ethical and a moral obligation to act as the patient's advocate in different caring situations. We want to point out that nurses should also act as the parents’ advocate and thereby indirectly support the children during NRMP.

Furthermore the findings in this study demonstrate that nurses have different opinions about restraining children during NRMP, and whether this can be supportive or not. Arguably there is an ambiguity as to how this is expressed. It is consistent with previous research by Brenner, Parahoo, and Taggart (2007) and Karen (2010). The main difference is the focus on consent and the strength used to restrain. Supportive holding requires consent and no strength is necessary, but during restraint nurses act without consent from the child and a certain amount of strength is used. The limits of this are difficult to define (Karen, 2010; Royal College of Nursing, 2003). We want to emphasize that this reasoning is only applicable if the child is not in a life-threatening condition.

Other findings stress that supporting their child is a natural part of parental responsibilities. In other words parents should not use restraint or perform tasks other than which pertains to their role as parents. This is in line with McGrath, Forrester, Fox-Young, and Huff (2002), Pearch (2005) and Schechter et al. (2007), who discuss that parents have a normal protective intuition, and some parents therefore find it difficult to restrain their children. We thus maintain that nurses must be flexible in responding to how and what parents express so that this limit is not exceeded.

Studies emphasize (Brenner et al., 2007; Pearch, 2005) that there is a lack of knowledge and guidelines for nurses in how to perform restraint safely. This in turn can lead to physical harm for the nursing staff (Lambrenos & McArthur, 2003) and children respectively, and Llyod, Urquhart, Heard, and Kroese (2008) find that nurses experience negative emotions during this action. In a study by Ives and Melrose (2010), the nurses described a feeling of powerlessness when the children had to endure those trying NRMP. Similarly, the nurses in our study reflected upon the negative consequences of a difficult procedure for the children but also for themselves. We believe that one possibility is if nurses perceive the procedure as being difficult, they can make an excuse that the restraint “is the best for the child.” We thus state that it is fair to assume that these emotions may affect the nurses’ supporting ability and are something they must be made aware. It is therefore also fair to assume that this can be one reason why the nurses in this study exhibited a degree of ambiguity as to whether the restraint can be supportive or not. This study did not reveal if nurses received training in how children can best be held.

### Methodological issues

We believe that the result shows a deeper understanding of the phenomenon, supporting children during NRMP, from the perspective of nurses, by using RLR and a phenomenological analysis according to Dahlberg et al. (2008).

The findings are restricted to a pediatric care setting. Adding participants from outside a pediatric care unit may perhaps have contributed to additional findings pertaining to children receiving care in other care settings. Unfortunately we were only able to get respondents from the pediatric unit.

Furthermore, the nurses in the present study have long professional experience, and this could have had an impact on the result. The current study shows that nurses’ experiences can affect their ability to support children during NRMP. Moreover, our hopes had been to recruit an equal number of men and women to the study, but as pediatric care is dominated by women the respondents here were only women.

Throughout the analysis, different questions emerged that were discussed among the co-authors, with the purpose of trying to increase the validity of the study. Such questions included the authors’ ability to have a bridling attitude (Dahlberg et al., 2008) towards the phenomenon. The first author has worked for many years with children undergoing NRMP, which means having a great pre-understanding in the matter. Attempts were made to have a bridling attitude, by trying to do what Dahlberg and Dahlberg (2003) describes as *not to take the indefinite as definite*.

Knowledge from this study may contribute to increasing nurses’ ability to support and thus improve the experience for children who need to undergo various NRMP.

## Conclusion and clinical implications

Having to endure NRMP can be experienced by children in many different ways, and nurses must be sensitive and listen to each individual child and as far as possible help those children cope with these procedures. Consequently, the nurses need to be responsive so that they are able to balance what they do and say in their attempt to reach the child's lifeworld, with the aim of being supportive. This includes meeting the child in their world which best can be done by a number of supportive actions, in which the cornerstone is parental participation and conversation. Being responsive to the child's experience, age, and development are additional aspects that need to be considered. This is based on the nurses’ professionalism and expertise in meeting the child in his/her own world. In the professionalism there is also the requirement that NRMP must be performed and that nurses somehow work within “a given framework” that sets the limit for the action. Being supportive during NRMP thus means that the nurses must see each child and thus decide on supportive actions and at the same time balance their responsibility for the completion of the procedure. For that to happen, the work can be described as “balancing on a tightrope” in an unpredictable situation.
